# Mixtures of Conditional Gaussian Scale Mixtures Applied to Multiscale Image Representations

**DOI:** 10.1371/journal.pone.0039857

**Published:** 2012-07-31

**Authors:** Lucas Theis, Reshad Hosseini, Matthias Bethge

**Affiliations:** 1 Werner Reichardt Centre for Integrative Neuroscience, Tübingen, Germany; 2 Max Planck Institute for Biological Cybernetics, Tübingen, Germany; 3 Bernstein Center for Computational Neuroscience, Tübingen, Germany; National Taiwan University, Taiwan

## Abstract

We present a probabilistic model for natural images that is based on mixtures of Gaussian scale mixtures and a simple multiscale representation. We show that it is able to generate images with interesting higher-order correlations when trained on natural images or samples from an occlusion-based model. More importantly, our multiscale model allows for a principled evaluation. While it is easy to generate visually appealing images, we demonstrate that our model also yields the best performance reported to date when evaluated with respect to the cross-entropy rate, a measure tightly linked to the average log-likelihood. The ability to quantitatively evaluate our model differentiates it from other multiscale models, for which evaluation of these kinds of measures is usually intractable.

## Introduction

Probabilistic models of natural images are used in many fields related to vision. In computational neuroscience, they are used as a means to understand the structure of the input to which biological vision systems have adapted and as a basis for normative theories of how those inputs are optimally processed [1], [2]. In computer science, they are used as priors in applications such as image denoising [3], compression [4], or reconstruction [5], and to learn image representations that can be used in object recognition tasks [6]. The more abstract goal common to these efforts is to capture the statistics of natural images.

The dominant approach to modeling whole images has been to use undirected graphical models (or *Markov random fields*). This is despite the fact that directed models possess many advantages over undirected models [5], [7]. In particular, sampling as well as exact maximum likelihood learning can often be performed efficiently in directed models while presenting a major challenge with most undirected models. Another problem faced by undirected models is the question of how to evaluate them. Ideally, we would like to quantify the amount of second- and higher-order correlations captured by a model. For stochastic processes, this can be done by calculating the cross-entropy rate between the learned distribution and the true distribution. However, the cross-entropy rate is typically difficult to estimate in undirected models so that these models are often evaluated only with respect to simple statistics computed from model samples or simply based on the samples' visual appearance. These measures, however, are less objective and hence need to be used with great caution. A large lookup table storing examples from the training set, for example, will reproduce samples which are indistinguishable from true image samples. Yet this model effectively assigns zero probability to images that have not been stored in the lookup table and would perform miserably if evaluated based on the cross-entropy rate. Evaluation of the cross-entropy rate is therefore crucial for the comparison of natural image models and an important step in measuring the progress which has been made in capturing the statistics of natural images.

Following the directed approach, we will demonstrate here that a directed model applied to multi-scale representations of natural images is able to learn and reproduce interesting higher-order correlations. We use multiscale representations to separate the coarser components of an image from its details, thereby facilitating the modeling of both very global and very local image structure. The particular choice of our representation makes it possible to still evaluate the cross-entropy rate.

## Methods

**Figure 1:**
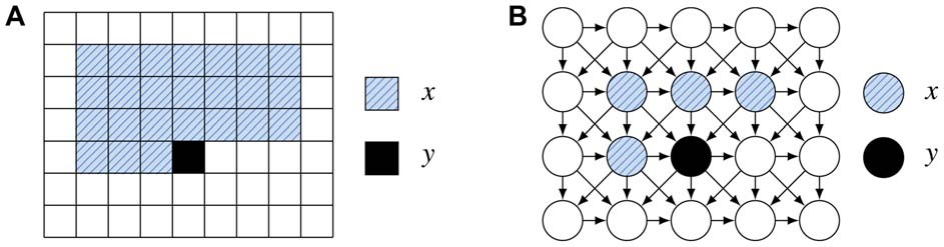
Directed image modeling. (A) A conditional model with a twenty-four pixel causal neighborhood. Sampling is performed by shifting the causal neighborhood from left to right and from top to bottom. (B) A graphical model representation with only four pixels in the causal neighborhood. The parents of a pixel are constrained to pixels which are above of it or in the same row and left of it.



One way to model the statistics of arbitrarily large images is to use a directed model in which the parents of a node are constrained to pixels which are left or above of it (as in Figure 1). A set of parents fulfilling this constraint is also called a *causal neighborhood* [7]. Note that a pixel will still depend on neighbors in all directions, that is, the causal neighborhood assumption puts only mild constraints on the size or shape of a pixel's Markov blanket. An advantage of the directed model is that it allows us to easily decompose the distribution defined over images or, more generally, a two-dimensional stochastic process 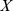 indexed by 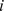 and 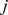, into a product of conditional distributions:

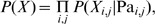
(1)where 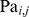 refers to the causal neighborhood of pixel 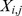. Consequently, performing maximum likelihood learning by maximizing the log-likelihood of the model can be done by optimizing a set of conditional probability distributions. An image is sampled from the model by shifting the causal neighborhood from top to bottom and from left to right, filling an image row by row. This procedure requires that the top rows and left columns of the image are initialized somehow to provide input to the conditional distributions. As a consequence, only after the procedure has generated a few rows and converged to the distribution of the model will it generate the desired samples.

### Mixture of conditional Gaussian scale mixtures

To complete the model, the conditional distribution of each pixel given its causal neighborhood has to be specified. We will assume stationarity (or shift-invariance), so that this task reduces to the specification of a single conditional distribution. A family of distributions which has repeatedly been shown to contain suitable building blocks for modeling the statistics of natural images is given by *Gaussian scale mixtures* (GSMs) 8,9,
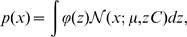
(2)where 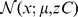 is a multivariate Gaussian density with mean 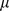 and covariance 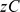, and 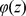 is any univariate density over scales 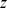. Mixture models and Markov random fields based on GSMs have been successfully applied to denoising tasks [3], [10]. When used in the directed setting also employed here, GSMs have been shown to yield highly improved estimates of the multi-information rate of natural images [7].

Here we use the conditional distribution of a *mixture of GSMs* to model the distribution of a pixel given its causal neighborhood. We restrict ourselves to mixtures of finite GSMs, that is, GSMs with a finite number of scales, and to mixtures in which each component and scale has equal a priori weight. Additionally, we assume that each GSM has mean zero. If variables 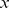 and 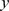 are modeled jointly with a mixture of GSMs, the conditional distribution of 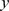 given 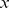 can be written as
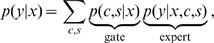
(3)where 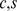 run over mixture components and scales, respectively. From the formulation in Equation 3 it is clear that the conditional distribution falls into the *mixtures of experts* framework [11]. In this framework, the predictions of multiple predictors – the *experts* – are mixed according to weights which are computed locally by the *gates*. For mixtures of GSMs, we have



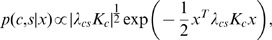
(4)

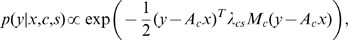
(5)


where 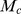 and 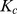 are positive definite matrices and the scales 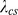 are positive real parameters. The gates provide a weighting of the different experts based on the covariance structure and scale of the input variables 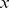. Each expert is a Gaussian with a certain covariance and a mean linearly predicted by the matrix 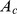. The conditional distribution can equivalently be described as a mixture of conditional Gaussian scale mixtures (MCGSM), because conditioned on 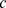, the conditional distribution becomes a conditional GSM [7].

### A simple multiscale representation

**Figure 2:**
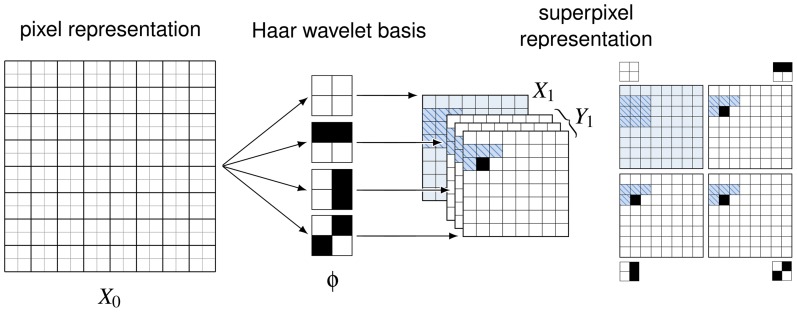
A multiscale image representation. Starting with a regular gray-scale image, the pixels are grouped into two by two pixels. Each group is then transformed using the Haar wavelet basis on the right. The resulting basis coefficients can be interpreted as channels of an image of which one channel represents the low-pass information and the other channels represent high-pass information. Just as in the original representation, we can define a directed model and causal neighborhoods for the superpixel representation. If the low-resolution image is given, the prediction of a pixel can be based on information from anywhere in the low-resolution image (not just a causal neighborhood) without losing the ability to efficiently sample or optimize the parameters of the model.

To facilitate the modeling of global as well as local structure, we introduce a multiscale representation which allows us to generate images by first sampling a low resolution image at the coarsest level and then iteratively adding more and more levels of increasingly finer scale. For simplicity, we will use the Haar wavelet representation. Before explaining the generative model which proceeds from coarse to fine, we recapitulate how the Haar wavelet coefficients can be obtained for a given image by transforming it iteratively proceeding from finer to coarser levels. For each iteration, the transformation is obtained as follows: The pixels of an image are first grouped into 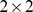 pixel blocks. Each block is then transformed using the orthogonal Haar wavelet basis (Figure 2). One component of the Haar basis, also called the DC component, essentially performs a block-average. The other three AC components encode the remaining details of the image. In this way one obtains four smaller images which together contain the full information about the original image. Subsequently, the same procedure can always be applied to the low resolution image again.

**Figure 3:**
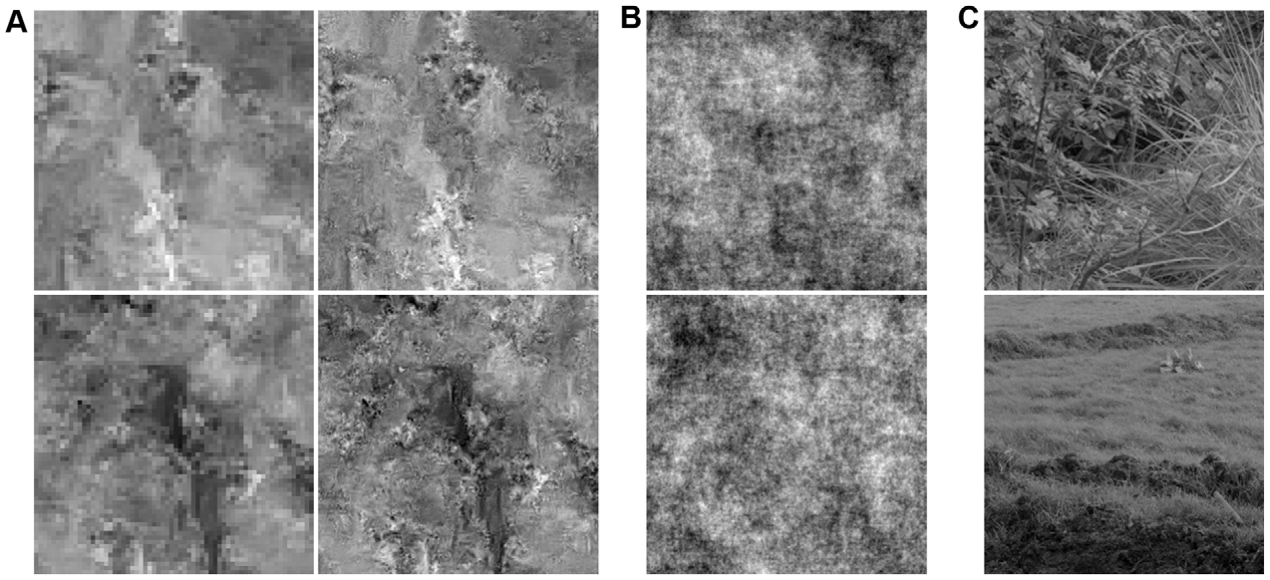
Samples from model trained on natural images. (A) To visualize the contribution of the different MCGSMs at the different scales, the first column shows samples from the MCGSM at the largest scale (low resolution). This sample was obtained using the top layer single-scale MCGSM. The second column shows samples from the full model, conditionally sampled with respect to the sample on the left. These samples therefore also contain the high-resolution information. The image on the left can be recovered from the image on the right through block-averaging. (B) The third column shows the same samples with all higher-order correlations destroyed but the autorocorrelation function left intact. This shows that the characteristic features of our samples are due to learned higher-order correlations and that the second-order correlations of natural images are faithfully reproduced as well. (C) For comparison, the right most column shows examples of images from the training set [14].

**Figure 4:**
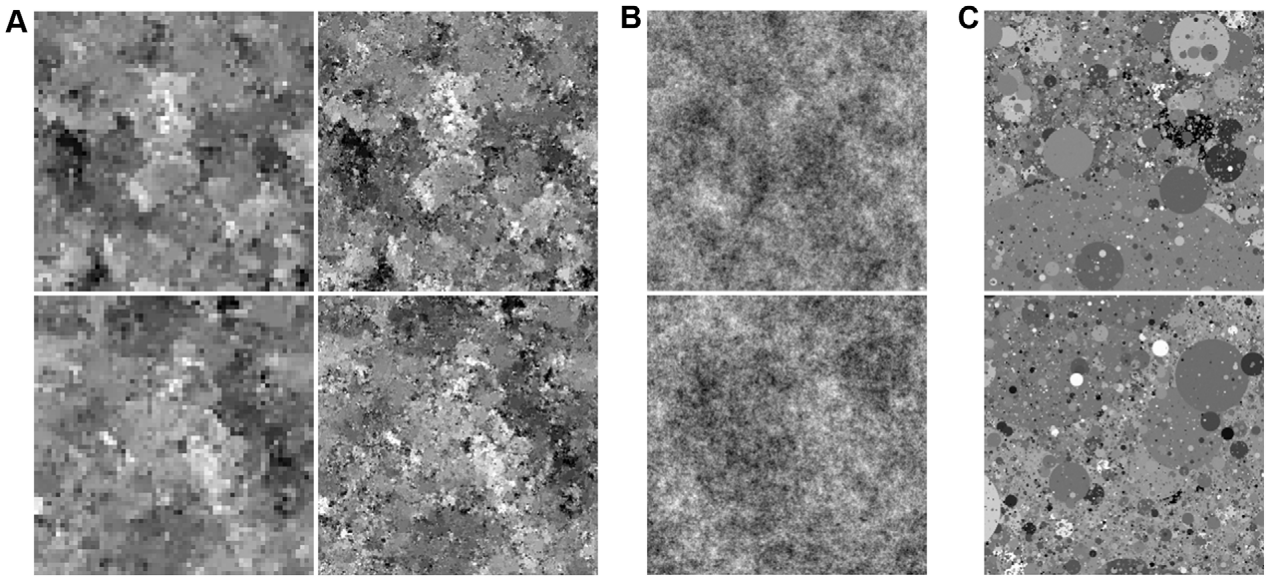
Samples from model trained on dead leave images. (C) The model was trained on samples from an occlusion-based model [17]. Example images from the training set are given on the right. (A) As above, the first two columns show samples from our model at two different scales. (B) The third column shows the same samples with all higher-order correlations destroyed, revealing second-order statistics which are very similar to the ones learned from natural images.

**Figure 5:**
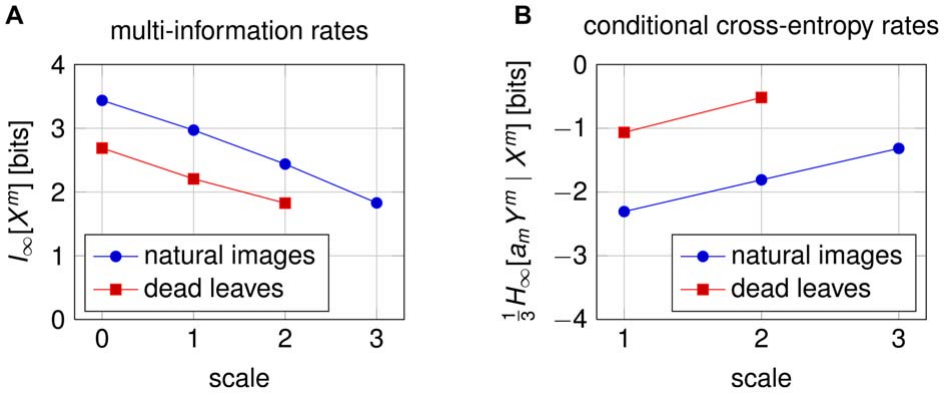
Multi-information and cross-entropy rates. (A) The estimated multi-information rate decreases steadily as the scale increases (the resolution decreases). (B) The conditional cross-entropy rate increases with scale. The factor 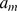 corrects for the change in variance due to block-averaging and can be different for each scale 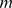. This shows that the van Hateren dataset [14] is generally not scale-invariant. A very similar behavior is shown by images created with an occlusion based model [17].

**Table 1:** Multi-information rate estimates.

model	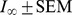 [bit/pixel]
MCGSM+multiscale	3.44±4E-3
MCGSM	3.40±4E-3
CGSM	3.26±5E-3
MCG	3.25±4E-3
CG (Gaussian)	2.70±7E-3

Multi-information rate (MIR) estimates of natural images obtained with different models including the conditional Gaussian scale mixture (CGSM) with a 7x7 causal neighborhood [7] and a mixture of conditional Gaussians (MCG) with a 5x5 causal neighborhood [5]. The SEM corresponds to one standard deviation of the estimate for different test sets. Since each model gives us a lower bound on the true MIR, a larger value corresponds to a better model.

**Figure 6:**
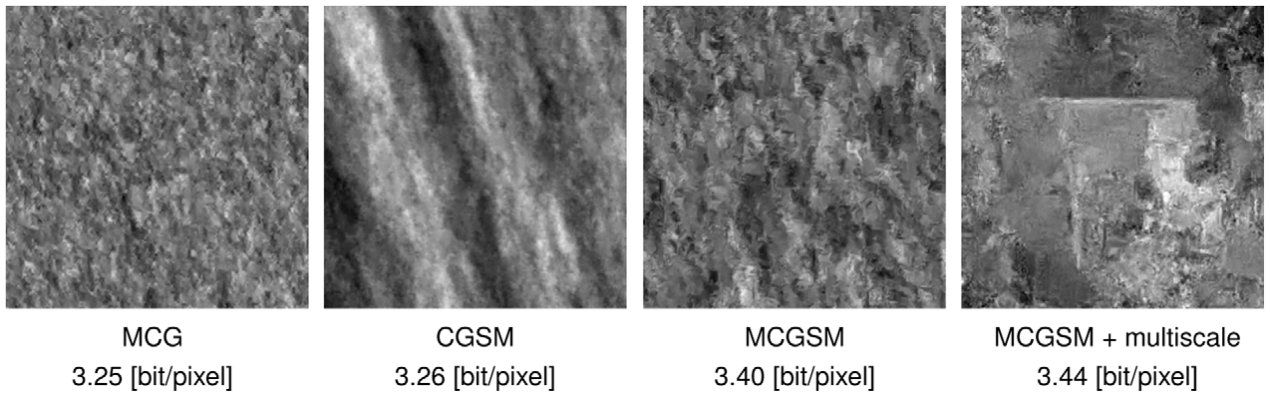
Natural image samples from different models. From left to right: Samples from a mixture of conditional Gaussians [5] (5×5 neighborhoods, 5 components including means), a conditional Gaussian scale mixture [7] (7×7 neighborhoods, 7 scales), a mixture of conditional Gaussian scale mixtures and the multiscale model. The appearance of the samples changes substantially from model to model.

**Figure 7:**
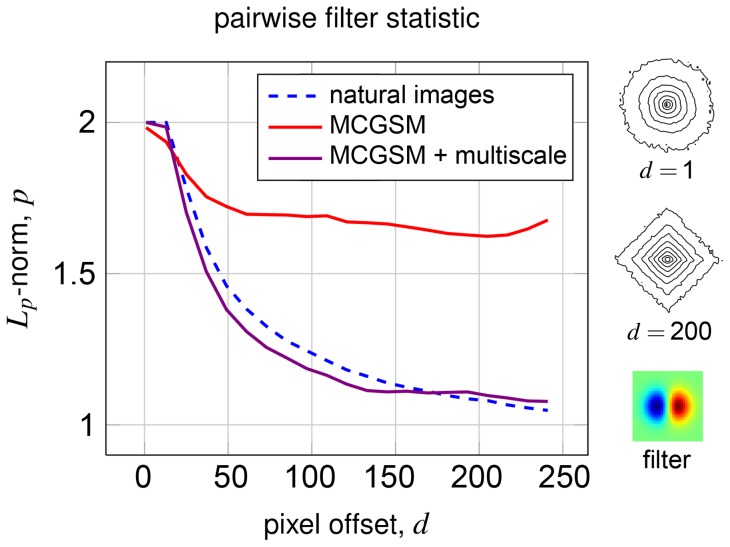
Pairwise filter statistics. The joint histogram of pairs of Gaussian derivative filter responses changes as their spatial separation increases. 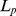-spherically symmetric distributions were fitted to the filter responses for natural and synthetic data. The vertical axis shows a maximum likelihood estimate of the parameter 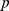. The horizontal axis shows the vertical offset between the position of the two filters. The plot shows that the multiscale representation enables our model to match the statistics of pairwise filter responses over much longer distances, which could be one possible explanation for the better performance in terms of the cross-entropy rate.

Since the four images obtained from each iteration of the wavelet transform all share the same topology, one can also view them as an image with multiple channels just like there are three different color channels at each pixel location for color images. We refer to a group of four coefficients at one location in the new representation as a *superpixel*. Similarly to the generative model defined in Equation 1 and illustrated in Figure 1, we could model images in this new representation with an MCGSM which tries to predict all channels of a superpixel at once, given a causal neighborhood of superpixels.

The essential difference when building a multiscale generative model that iteratively proceeds from coarse to fine is to assume at each level that the DC channel has already been specified by the previous iterations and only the remaining three AC channels need to be predicted. Importantly, this implies that the restriction to a causal neighborhood only persists for the AC channels but does not apply to the DC channel anymore. In other words, we can now base our predictions on an arbitrary set of pixels from the low-resolution image (that is, the DC channel) which is not confined to a causal neighborhood. If 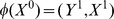 is the superpixel representation of an image 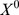 with a low-resolution (DC) part 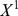 and a high-resolution (AC) part 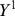, we have
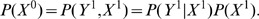
(6)


The same decomposition can be applied again to 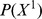, then again to 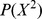, and so on. That is, we will model images in this representation using the following factorization:
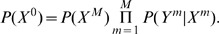
(7)


Due to this factorization, we can sample an image by first sampling a low-resolution image 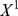 and then conditionally sampling 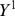. Each factor on the right-hand side again factors into a product of the form of Equation 1,
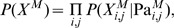
(8)

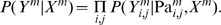
(9)


Every variable that has already been sampled can be used to conditionally sample all other variables. In this way, we obtain a complete set of Haar wavelet coefficients. To reconstruct an image from the Haar wavelet coefficients, we start with the low-resolution image at the coarsest scale, 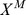, and merge it with the AC coefficients at the next level, 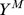, to give a higher-resolution image 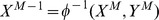. We repeat this process until we obtain the image at the original resolution, 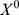.

In the following, we will model the distributions 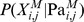 and 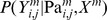 using MCGSMs (Equations 3 to 5). We will use a different density for each scale 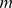, but the same density for all locations 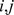 within one scale. Maximum likelihood learning in this case amounts to learning to predict 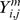 from 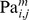 and 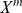, and 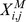 from 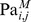 by maximizing the average log-likelihood of each conditional density. Maximizing the likelihood of the transformed image is equivalent to maximizing the likelihood of the original image because our Haar transform is just an orthogonal transformation. Otherwise we would have to take into account the transform's Jacobian determinant.

### Model evaluation

A principled way to evaluate a model approximating a stochastic process 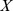 is to use the model for estimating the true distribution's *multi-information rate* (MIR) [7], [12],
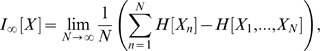
(10)where 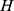 denotes the (differential) entropy. A related measure is the *entropy rate* [13].



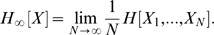
(11)For a strictly stationary Markov process, one can show that these quantities reduce to [7], [13]

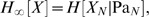
(12)

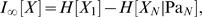
(13)


for some 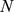. The *cross-entropy* between a target distribution and any approximating model distribution with density 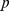 is defined here as 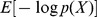, where the expectation is with respect to the target distribution. Analogous to the entropy rate (Equation 11), we can define the *cross-entropy rate* as a limit of cross-entropies. It is readily shown that the cross-entropy rate is equal to 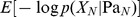, where 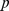 is now a model density approximating the distribution of 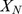 given 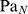. By replacing the entropy rate with the cross-entropy rate in Equation 13 (second term), we obtain a lower bound on the true MIR. In the following, we will call this lower bound the *cross-MIR*.

If the assumption of stationarity or the Markov assumption is not met by the true distribution, the cross-MIR will still be a lower bound but will become less tight [7]. The difference between the true MIR and the cross-MIR is the Kullback-Leibler divergence between the true distribution and the model distribution. Therefore, the better the approximation of the model distribution to the true distribution, the larger the cross-MIR.

Maximizing the cross-MIR by minimizing the cross-entropy rate is the same as maximizing the average log-likelihood of the conditional distributions. The MIR quantifies the amount of second- and higher-order correlations of a stochastic process. Similar to the likelihood, the cross-MIR can be said to quantify the amount of correlations captured by a model. In addition, it has the advantage of being easier to interpret than the likelihood or the cross-entropy rate, as it is always non-negative and invariant under multiplication of the data with a constant factor. An independent white noise process has a MIR of zero. In the stationary case, evaluating the cross-MIR amounts to calculating one marginal entropy and one conditional cross-entropy (Equation 13).

Since the superpixel representation is just a linear transformation of the original image, we can evaluate the entropy rate also for the multiscale model. Using the fact that the transformation has a Jacobian determinant of 1, the following relationship holds for both entropy and cross-entropy rates:
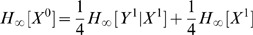
(14)

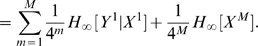
(15)


The factor 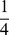 is due to the superpixel representation having four channels. In order to estimate the cross-entropy rate of our model, we only need to compute the cross-entropy rates at the different scales and form a weighted average.

## Results

### Natural images

We extracted training data at four different scales from log-transformed images taken from the van Hateren image dataset [14]. In all experiments, we used 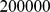 training examples of inputs and outputs.

To model the coarsest scale, we used an MCGSM with a causal neighborhood corresponding to the upper half of a 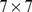 neighborhood surrounding the predicted pixel (as in Figure 1). For the finer scales, we trained three MCGSMs with 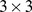 superpixel neighborhoods (as in Figure 2; using the full neighborhood and not only the upper half). All models were comprised of 8 components with 4 scales each. We found that first-order optimization methods performed poorly compared to second-order optimization methods in tuning the model's parameters. For second-order optimization, we used the quasi-newton method BFGS [15] (gradients of the parameters are provided in Appendix S1). The small patch sizes were chosen mainly for computational reasons. Note that the number of parameters grows as 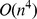 for 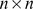 neighborhoods (because of the gating covariance matrices, Equation). Since the time and space complexity of BFGS grows quadratically with the number of parameters, or as 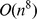, using larger patches was computationally prohibitive. For faster convergence, we initialized the conditional models with parameters from mixtures of GSMs trained on the joint distribution of inputs and outputs using expectation maximization [16].

To sample from the model, we first generated an image using the single-scale MCGSM at the coarsest scale. We initialized the boundaries of the image sample with small Gaussian white noise and then sampled images by sequentially sampling each pixel from left to right and top to bottom. The images were large enough to allow the sampling procedure to converge to the model's stationary distribution. After sampling a large image, we extracted its center part and used it as input to the model at the next finer scale. The sampling procedure converged quickly and the choice of initialization was therefore noncrucial. Using true natural images for initializing the boundaries yielded similar results.

Samples from the model are shown in Figure 3A. We find that the model is able to generate images with some interesting properties that cannot be found in samples of other models of natural images. Perhaps the most striking property of the sampled images is the heterogeneity expressed in the combination of flat image regions with regions of high variance as it can also be observed in true natural images.

By destroying the higher-order correlations in the samples while keeping the second-order correlations intact, we obtain the familiar pink noise images (Figure 3B). This shows that the model faithfully reproduces the autocorrelation function of natural images, and that the characteristic features of the sampled images are due to higher-order correlations learned by the model. The higher-order correlations were removed by replacing the phase spectrum of the image with a random phase spectrum obtained from a white noise image but keeping the sample's amplitude spectrum. For stationary processes, the amplitude spectrum defines the autocorrelation function of an image and vice versa.

### Dead leave images

As a further test, we generated a more controlled dataset with 1000 images of size 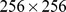 pixels sampled from an occlusion model (“*dead leaves*”) using the procedure described by Lee and Mumford in [17]. Afterwards, we added small Gaussian white noise to the samples as without the noise, the multi-information rate would be infinite. The dead leave model was designed to generate samples which are approximately scale invariant and share many properties with natural images. In particular, dead leave images share very similar marginal and second-order statistics with natural images. Many of the difficult-to-capture higher-order correlations found in natural images are also believed to be caused by occlusions in the image. This dataset should therefore pose similar challenges as the set of natural images. We extracted training data at three different scales and used the same neighborhood sizes and the same training procedure as above. Samples generated by our model are shown in Figure 4A. Clearly, our model has not learned what a circle is. However, it is able to reproduce the blotchiness of the original samples despite having no built-in knowledge of occlusions.

### Scale invariance

The multiscale representation lends itself to an investigation of the scale invariance property of natural images. The statistics of a scale-invariant process are invariant under block-averaging and appropriate rescaling to compensate for the loss in variance [18]. Using the notation as above, this would mean that 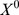 is distributed as 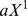 for some 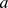. This in turn implies that the multi-information rate (MIR) should stay constant as a function of the scale. Because the MIR is invariant under rescaling with a constant factor, we can ignore the rescaling factor 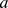.

We estimated the multi-information rate of the van Hateren dataset with the cross-MIR of our model (Figure 5). The cross-entropy rates were calculated as in Equation 15.

Scale-invariance of natural images is typically tested by looking at simple statistics such as the distribution of certain filter responses. While these statistics can be surprisingly stable across scales, the steady decrease of the information rate suggests that the van Hateren natural image dataset is not very scale-invariant. For example, a consequence of a smaller MIR at larger scales is that pixels become more difficult to predict from neighboring pixels. However, the difference in cross-MIR could also be caused by the fact that we are using a slightly different model at the largest scale than for modeling the image details at the lower scales. This problem is not shared by the conditional entropy rates plotted on the right of Figure 5, because each conditional entropy depends only on a single model. If the images were indeed scale invariant, the distribution over 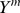 and 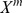 should not change with scale 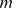, subject to proper rescaling. Since we are using the same model (but with separately learned parameters) to model the relationship between 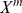 and 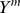 for all 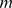, the estimated entropy rates should stay constant even if our model performed poorly. Our results are consistent with the findings of Wu et al., who showed that many natural images are more difficult to compress at larger scales and argued that the entropy rate of natural images has to increase with scale [19]. We find a similar drop in MIR for dead leave images. Since these images were designed to be as scale-invariant as possible, this shows that our model and the MIR are very sensitive to these differences in statistics.

### Multi-information rates

Using an estimate of the marginal entropy of 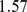 bits [7], we arrive at an estimated multi-information rate of 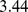 bits per pixel for the van Hateren dataset (Table 1). This is approximately 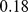 bits better than the current best estimate for natural images [7] and 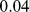 bits better than our result obtained without using the multiscale representation. Note that even for a small image of 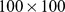 pixels, differences of 0.04 and 0.18 bit per pixel give rise to absolute differences of 400 and 1800 bits, respectively.

Since the true MIR of natural images is unknown, this increase in performance does not tell us how much closer we got to capturing all correlations of natural images. It also does not reveal in which way the model has improved compared to other models. However, samples and statistical tests can give us an indication. Figure 6 shows samples drawn from models suggested by Domke et al. [5], Hosseini et al. [7], and samples from the models presented in this paper. The substantial change in the appearance of the samples suggests that the increase from 3.40 bits to 3.44 bits reflects a meaningful improvement.

Another way to demonstrate an improvement is to investigate sample-based test statistics. The joint statistics of the responses of two edge filters applied at different locations in an image are known to change in certain ways as a function of their spatial separation and are difficult to reproduce [20]. We apply a vertically oriented Gaussian derivative filter at two vertically offset locations and record their responses (for a more detailed explanation, see Appendix S2). After whitening, the filter responses are approximately 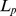-spherically symmetric. We therefore fit an 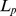-spherically symmetric model [21] with a radial Gamma distribution to the responses and, at every distance, record the parameter 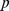 of the model's norm. Since the marginal distribution of each filter response is highly kurtotic and the responses become more independent as the filter distance increases, the joint histogram becomes more and more star shaped. This is expressed in the optimal value for 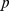 becoming smaller and smaller. As plotted in Figure 7, the behavior of the optimal 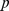 is not well reproduced using a single scale but is captured by our multiscale model.

## Discussion

We have shown how to use directed models in combination with multiscale representations in a way which allows us to still evaluate the model in a principled manner. To our knowledge, this is the only multiscale model for which the likelihood can be evaluated. Despite the model's computational tractability, it is able to learn interesting higher-order correlations from natural images and yields state-of-the-art performance when evaluated in terms of the multi-information rate. In contrast to the directed model applied to images at a single scale, the model also reproduces the pairwise statistics of filter responses over long distances. Here, we only used a simple multiscale representation. Using more sophisticated representations might lead to even better models. For reasons explained above, the neighborhood sizes used by our models were still fairly small. This is a problem which could be solved in future implementations using different parametrizations or optimization methods.

Code for training and evaluating MCGSMs on multiscale image representations can be found at http://bethgelab.org/code/theis2012/.

## Supporting Information

Appendix S1 **Details on the parametrization and gradients of the conditional log-likelihood** (**Equation 3**)**.**
(PDF)

Appendix S2 **A more detailed explanation of how to generate** **Figure 7** **.**
(PDF)
