# Supplementary material for: Mixtures of Conditional Gaussian Scale Mixtures Applied to Multiscale Image Representations
Source: PLoS One. 2012 Jul 31;7(7):e39857. doi: 10.1371/journal.pone.0039857 (PMC3409213; doi:10.1371/journal.pone.0039857)
Supplement: Appendix S1 — Details on the parametrization and gradients of the conditional log-likelihood ( Equation 3 ). (PDF) [file pone.0039857.s001.pdf]

Appendix S1

In the following, we list the gradients necessary for implementing our model. For any parameter θ, the derivative of the conditional log-likelihood (Equation 3, main text) is

$$\frac{\partial}{\partial\theta}\log p(y \mid x, \theta) = \sum_{c,s} p(c, s \mid x, y)\frac{\partial}{\partial\theta}\log p(c, s, y \mid x) \tag{1}$$

where

$$\frac{\partial}{\partial\theta}\log p(c, s, y \mid x) = \frac{\partial}{\partial\theta}\log p(c, s \mid x) + \frac{\partial}{\partial\theta}\log p(y \mid c, s, x). \tag{2}$$

The first term can be written as

$$\frac{\partial}{\partial\theta}\log p(c, s \mid x) = \frac{\partial}{\partial\theta}\log\frac{f(c, s; x)}{\sum_{c,s} f(c, s; x)} \tag{3}$$

$$= \frac{\partial}{\partial\theta}\log p(c, s, x) - \sum_{c,s} p(c, s \mid x)\frac{\partial}{\partial\theta}\log f(c, s; x), \tag{4}$$

where $f(c, s; x)$ (the *gate*) is defined as in Equation 4 of the main text to be the unnormalized conditional probability

$$f(c, s; x) = |\lambda_{cs}|^{\frac{N}{2}}|L_c|\exp\left(-\frac{1}{2}x^\top\lambda_{cs}L_cL_c^\top x\right). \tag{5}$$

We replaced the precision matrices K_c and M_c (Equations 4 and 5, main text) by their Cholesky decompositions $L_cL_c^\top$ and $B_cB_c^\top$, respectively, and optimized over the Cholesky factors instead. The conditional density $p(y \mid c, s, x)$ (the *expert*) is Gaussian and therefore easily normalized,

$$p(y \mid x, c, s) = \frac{\lambda_{cs}^{\frac{M}{2}}}{(2\pi)^{\frac{M}{2}}}|B_c|\exp\left(-\frac{1}{2}(y - A_cx)^\top\lambda_{cs}B_cB_c^\top(y - A_cx)\right). \tag{6}$$

What is left is to compute the gradients of $\log p(y \mid x, c, s)$ and $\log f(c, s; x)$,

$$\frac{\partial}{\partial L_c}\log f(c, s; x) = \text{diag}(L_c)^{-1} - \lambda_{cs}xx^\top L_c, \tag{7}$$

$$\frac{\partial}{\partial\lambda_{cs}}\log f(c, s; x) = \frac{N}{2}\lambda_{cs}^{-1} - \frac{1}{2}x^\top L_cL_c^\top x, \tag{8}$$

$$\frac{\partial}{\partial\lambda_{cs}}\log p(y \mid c, s, x) = \frac{M}{2}\lambda_{cs}^{-1} - \frac{1}{2}(y - A_cx)^\top B_cB_c^\top(y - A_cx), \tag{9}$$

$$\frac{\partial}{\partial B_c}\log p(y \mid c, s, x) = \text{diag}(B_c)^{-1} - \lambda_{cs}(y - A_cx)(y - A_cx)^\top B_c, \tag{10}$$

$$\frac{\partial}{\partial A_c}\log p(y \mid c, s, x) = \lambda_{cs}B_cB_c^\top(y - A_cx)x^\top. \tag{11}$$

Here, M is the dimensionality of y ($M = 3$ for the multiscale MCGSM and $M = 1$ for the regular MCGSM) and N is the dimensionality of the neighborhood x. The function diag returns a matrix with all off-diagonals set to zero, $\text{diag}(M)_{ij} = \delta_{ij}M_{ij}$.

We tried regularizing the parameters by specifying different prior distributions for the parameters and optimizing the posterior probability of the parameters instead of their likelihood. However, we found no improvement in the model's performance. We also tried adding means to the gates and experts, which also did not help to improve the performance of the model when evaluated on natural images.
