# Supplementary material for: Mixtures of Conditional Gaussian Scale Mixtures Applied to Multiscale Image Representations
Source: PLoS One. 2012 Jul 31;7(7):e39857. doi: 10.1371/journal.pone.0039857 (PMC3409213; doi:10.1371/journal.pone.0039857)
Supplement: Appendix S2 — A more detailed explanation of how to generate Figure 7 . (PDF) [file pone.0039857.s002.pdf]

Appendix S2

To generate Figure 7 of the main text, we used a vertical Gaussian derivative filter,

$$f(x,y;\sigma) = \exp\left(-\frac{x^2+y^2}{2\sigma^2}\right)x, \tag{1}$$

where in our experiments $\sigma = 0.5$. We repeatedly applied the filter at vertically offset locations (x, y) and $(x, y+d)$ with a fixed distance d and recorded a pair of filter responses $z \in \mathbb{R}^2$ at each location. The locations were picked uniformly at random. For each d, we modeled the distribution of filter responses using an L_p-spherically symmetric distribution [1], while using a Gamma distribution to model the radial component $||z||_p$. The density of the distribution is given by

$$g(z;p,k,\theta) = \frac{p^{n-1}\Gamma(n/p)}{2^n\Gamma(1/p)^n}\frac{1}{||z||_p^{n-1}}\gamma(||z||_p;k,\theta) \tag{2}$$

$$= \frac{p^{n-1}\Gamma(n/p)}{2^n\Gamma(1/p)^n}\frac{1}{||z||_p^{n-1}}\frac{1}{\theta^k}\frac{1}{\Gamma(k)}||z||_p^{k-1}\exp\left(-\frac{||z||_p}{\theta}\right). \tag{3}$$

Γ is the gamma function, $\gamma(r;k,\theta)$ is the density of the Gamma distribution with shape parameter k and scale parameter θ and $n = 2$ is the dimensionality of z. Using maximum likelihood learning, we fitted all three parameters to the distribution of filter responses before recording the parameter p of the L_p-norm.

References

[1] Gupta AK, Song D (1997) L_p-norm spherical distribution. Journal of Statistical Planning and Inference 60: 241–260.
